# The Hospital. Nursing Section

**Published:** 1902-11-08

**Authors:** 


### The Hospital.

### Nursing Section.

Contributions for this Section of "THE HOSPITAL" should be addressed to the EDITOR, "THE HOSPITAL" NURSING SECTION, 28 & 29 Southampton Street, Strand, London, W.C.

No. 841.—VOL. XXXIII. *SATURDAY, NOVEMBER* 8, 1902.

### Notes on News from the Nursing World.

### HONOURS FOR ARMY SISTERS.

THE Honours List, published in the *Gazette*, issued by the War Office on Friday last, includes the names of several nurses whose services in South Africa the King has been graciously pleased to recognise by the bestowal of the decoration of the Royal Red Cross. They are as follows :—Superintendent Sister M. Russell and Nursing Sister C. H. Keer of the Army Nursing Service ; Nursing Sisters H. O. Luckie and Margaret Whiteman, of the Army Nursing Service Reserve ; Miss Katherine Blanche Brereton and Mrs. Margaret Scott Fripp, of the Imperial Yeomanry Hospital ; Superintendent Sister M. Rawson, of Victoria ; Sister Georgina Pope, of Canada ; and Sisters Joan Charleson, Harriet Maud Campbell-Ross, Ellena Philipson Stow, Isabella Jessie Philipson Stow, Amy Blake Knightley, Sophia Margaret Paterson, Katherin Louisa Hill, and Mrs. Sophie Lees, of Intombi Hospital Camp. Miss Whiteman, who was trained at Horton Infirmary, Banbury, and St. Bartholomew's Hospital, Rochester, has had a varied experience as charge nurse at Rotherham Hospital, head nurse at Great Yarmouth Hospital, and private nurse attached to Princess Christian's Trained Nurses' Home at Windsor. She nursed during the typhoid epidemic at Maidstone, and was awarded the medal. Miss Brereton was trained at Guy's Hospital, where she was afterwards sister, and she has also been staff nurse at Birkenhead Children's Hospital. Miss Rawson was trained at Leicester Infirmary and Rotunda Hospital, Dublin. In 1893 she went to Melbourne, and was first charge nurse and then sister in charge of the midwifery ward at the Women's Hospital in that city. She has also done private nursing in Kalgoorlie, Western Australia. Miss Paterson was trained at the Royal Infirmary, Glasgow, and, proceeding to South Africa, became staff nurse at the Provincial Hospital, Port Elizabeth ; and subsequently matron at the Addington Hospital, Durban.

### QUEEN ALEXANDRA'S IMPERIAL MILITARY NURSING SERVICE.

THERE have been criticisms in our columns in the regulations for admission to Queen Alexandra's Imperial Military Nursing Service which are distinctly worthy of notice and merit the attention of the authorities. But it must be remembered that the service is not yet fully organised, and that the regulations are by no means as unalterable as the law of the Medes and Persians. If it be found by the Nursing Board that in practice some of them do not work well, they will, we feel sure, be reconsidered, and either amended or withdrawn. For example, with regard to the criticism that the chances of the promotion of a nurse are only slender, we have reason to believe that the number of sisters, which is undoubtedly inadequate, will be materially increased. This would obviously improve the prospects of the staff nurses, and it would also tend to dispose of the objection that the sisters will have more than they can do. Concerning the question of control, if the sisters and staff nurses are given their proper place in the wards, we do not see why the scheme should not work well. The staff nurses, as we understand, will be treated as junior officers, and will be in a position above the orderlies. If the orderlies are not amenable to control, the sister will report them for punishment ; she will not be required, as one of our correspondents appears to imagine, to inflict it. In most warm climates there are so many native servants that even the orderlies have little to do in the shape of rough work. The staff nurses will practically, we conceive, have none at all. As to the delicate question of the social status of the sisters and staff nurses, it is essential that they should be women of refinement and education—gentlewomen in the truest sense of the word. The protest against the compulsory wearing of uniform on all occasions is not general, but it may be observed that there is a rule which gives discretionary power to a matron, and that no member of the service is likely to be required to cycle in uniform. There have been many inquiries respecting the non-inclusion of the Indian Nursing Service. We believe that no decision has yet been arrived at on this matter. Colonial nurses are eligible for admission if their parents are of British birth or a naturalised British subject, but not otherwise, and the Colonial Nursing Association is in no way affected by the formation of the new service. The Army Nursing Service Reserve still exists, but the process of disbanding the members called up for active service is proceeding. Neither members of the Army Nursing Service nor of the Reserve become members of Queen Alexandra's Imperial Military Nursing Service as a matter of course, but only if they are recommended by the Nursing Board as eligible. Their qualifications must be indisputable, and as to these no exception will under any circumstances be made. Those nurses who possess the indispensable training, and to whom the service offers attractions, may be interested to know that, although applications are numerous, the appointments are not all filled.

### LORD METHUEN'S COMPLIMENT.

TAKING the place of Princess Christian, who had promised to unveil a memorial to soldiers who fell in South Africa, at St. Stephen's Church, Bristol, on Monday, Lord Methuen delivered a brief address on the occasion. After alluding in warm terms to the military and civil surgeons "who had shown so much care and so much kindness to those who had been killed and to those who were wounded," he said that he also wished "to pay a tribute to the most beautiful of God's gifts to the soldier in active service—he meant the sisters and nurses who were with them." Out of the abundance of the heart the mouth speaketh, and the tribute of Lord Methuen will be all the more appreciated because it is well known that he himself had very considerable and prolonged experience which enabled him to measure the value of the services of the nurses in South Africa.

### A NEW STATION FOR ARMY SISTERS.

Sisters D. D. Tripp and C. Steen have received orders to hold themselves in readiness to embark shortly for Egypt to do duty in the Military Hospital at Khartoum. British troops are to form part of the permanent garrison of this rapidly developing town, and provision has therefore to be made for the sick; hence this new departure for the two sisters. Their term of duty will probably be for three years. The sisters selected have had several years of service, and both of them nursed in South Africa during the late war.

### CONTINUED FRICTION AT CROYDON INFIRMARY.

The difficulty of obtaining probationers at Croydon Workhouse Infirmary is illustrated by the statement of Mrs. Coburn-Coulton, the superintendent of nurses, which was embodied in a report of the Infirmary Committee of the Croydon Board of Guardians. The superintendent says that she provided six probationers with three dresses each, and, frankly admitting that she acted irregularly in the matter and expended the sum of £8 12s. 6d. without the authority of the board, she justifies her action on the ground that if she had not by this action secured the services of the probationers they would have gone elsewhere. The committee have advised the payment, but it has been intimated that the Board "will not again sanction such an irregularity." Other indications of the unsatisfactory condition of affairs at Croydon are that 23 of the nurses have asked the board for an inquiry into the present system of their training; and that the superintendent of nurses still refuses to carry out the rules as regards dining with the assistant matron and head nurses.

### PRIVATE NURSING IN SMYRNA.

From Smyrna, as well as from Egypt, comes a note of warning to those who think of undertaking private nursing abroad. A correspondent who has been for six years in that town writes us that no nurse who does not possess private means, and cannot speak French, ought not to think of going out from England. There are very few doctors who speak English, and most of the patients are Greeks, French, and Armenians. At present, she states, there are two English nurses engaged in private work in Smyrna, and they have plenty to do. But they both speak French and Greek, and have friends in the place. It is absolutely imperative that any nurse who proposes to take up her residence there should be a fully-qualified midwife, most of the cases being maternity. The pay in the English hospital is small, as the Smyrna people do not yet understand why a trained English nurse should receive so much more than a native woman.

### ASYLUM MATRONS.

Two letters in our issue of to-day from two different points of view upon the position of asylum workers are interesting reading. Dr. Urquhart, the medical superintendent of the James Murray Royal Asylum at Perth, whose experience and long connection with one of the best managed asylums in the United Kingdom, makes his opinion of value, strongly denies that "any asylum is competent to turn out nurses with the highest professional claims to appointments as matrons." He contends that "every matron ought to have proved her capabilities as a nurse; and that she should be qualified to undertake the care of all kinds of patients, and the instruction of the nurses in every detail." This means that, according to Dr. Urquhart, every matron of a hospital for the insane should be trained in a general hospital as well as in an asylum. He advises a nurse to begin in an asylum and thence to pass to a general hospital. As to "side door to promotion" by which nurses, trained in general, but not in mental, knowledge, gain the "higher berths in asylums," he prophesies that it will soon be closed. On the other hand, Mr. Charles Bridgeland of the Hants County Asylum, speaking from his own experience, maintains that "the training received in the infirmary ward of an ordinary public asylum is quite equal, if not superior, to that received in many of our large general hospitals and infirmaries." As far as male nurses are concerned this statement may be perfectly correct, but it certainly does not apply to female nurses. Mr. Bridgeland's suggestions as to how to remedy existing evils are, however, worth consideration.

### NURSES FROM SOUTH AFRICA.

The following nurses have arrived from South Africa during the past week:—On the *Orissa*, Sisters M. A. Turner, L. A. Clarke, M. Jephson, A. Barton, E. M. King, K. E. King, A. Cooper Lindsay, W. French, and L. O. Dixon, of the Army Nursing Service Reserve; on the *Simla*, S. E. Oram, S. I. Snowden, and Makepeace, of the Army Nursing Service, and N. P. H. Farrieria and J. H. Anderson, civil nurses; on the *Norman*, Sisters A. Beith and M. R. McDowell, of the Army Nursing Service Reserve; and on the *Nubia*, Sisters A. F. Clarke, A. M. Harrison, M. Hilson, J. McCotter, and A. G. Wason of the Army Nursing Service Reserve; and Sisters L. W. Tulloh, G. E. Chapman, M. Estcourt, and E. Timbrell of the Army Nursing Service, invalided.

### NURSES REMAINING IN SOUTH AFRICA.

We understand that several of the sisters who have been nursing at the hospital in Aliwal, on the borders of Cape Colony, are remaining in South Africa in order to take up private nursing in the new colonies. According to a recent visitor at the hospital, who had previously spent some time in the refugee camp, the number of patients has been reduced to eleven. This doubtless explains the fact that when the visitor arrived at the hospital the sisters were engaged in a game of tennis. Each sister has had five probationers under her selected from among the Boer girls in the camp. But we presume that this arrangement will shortly come to an end.

### NURSES IN COMIC OPERA.

An entertainment was given in the Devonshire ward of the Chesterfield and North Derbyshire Hospital and Dispensary by the nursing staff, assisted by a number of gentlemen, on October 29th and repeated on the 30th. The object was to help the funds of the hospital, and it succeeded remarkably well. The residents of the town and neighbourhood were present on the first night, and old patients and their friends the second, the ward being crowded on each occasion. The programme consisted of selections from "The Mikado" and "The Geisha," rendered in character. The costumes were admirable and were made by the nursing staff under the direction of the matron. The effective way in which the pieces were performed, showed how thoroughly the nurses had learned their respective parts. The stage scenery and the drapery helped to give a finish to the proceedings. The amount realised by the entertainments was £47.

### NURSING HOMES IN MARYLEBONE.

A very strong protest has been made by several of the residents in Marylebone against the multiplication of nursing homes in the parish. They complain that the pleasures of life are not enhanced by the "inhalation of noxious odours emanating from nursing homes; the hearing of groans from patients under operations; or by walking over a mass of malodorous and perhaps putrefying straw placed in the street for the pecuniary advantage of nursing institutions." A more serious plea is, however, that "there have been found in certain so-called nursing homes domestic servants and ex-barmaids, and, without hospital training, wearing nurses' costumes." "Nursing" homes in which persons who are not nurses are employed, would soon cease to exist if such places were periodically subjected to inspection. No other remedy but this and the compulsory registration of homes can be efficacious.

### SOMERSETSHIRE DISTRICT NURSES.

The second social gathering for Somerset District Nurses and others was held last week in the rooms of the Women's Total Abstinence Union at Weston-super-Mare. Two excellent addresses on "Temperance from a Nurses' Point of View" were given by Miss Green and Mrs. Parnell of the Memorial Hospital, Paulton. There were nearly twenty nurses present. It is hoped that the next meeting may be arranged for some time in January, and be held at Taunton, when the subject for discussion will be "The Feeding of Infants."

### FIFTEEN AND A HALF HOURS A DAY.

The reactionaries have been overruled at Chelmsford, although nine members of the Board of Guardians voted against the recommendations of the committee which we mentioned last week. The urgent need of acting upon them, as affirmed by the nineteen Guardians who constituted the majority, is shown from the remarks of Captain Hervey, the Local Government Board Inspector, who was asked by the Chairman to speak on the question. He reminded the Guardians that more than a year ago he had said that the nursing staff was totally inadequate, and asked whether they "knew that the nurses were working 15½ hours a day." At this point a member of the Board interjected the remark, "They have never complained"; but Captain Hervey effectively retorted that they had complained to him and to Dr. Fuller, the Medical Inspector of the Local Government Board, "over and over again." They, however, had urged the staff to stick to their duties, because they felt sure that the Guardians would see the necessity of doing something. And now, at last, we trust that the iniquity of working the nurses 15½ hours a day will cease.

### BIGOTRY AT GUILDFORD.

The question of the appointment of a matron of the Guildford and District Isolation Hospital was discussed last week by the local authorities. A committee of the Hospital Board recommended that the appointment should be advertised, and it was asked why they had not advised the appointment of Miss Healy, who was temporarily in charge. It then transpired that the only reason why she was ignored was "that the committee thought it was not desirable that a Roman Catholic should have charge of such an institution." The explanation not only provoked a strong protest on the ground that Miss Healy's religion ought not to have influenced the committee, but resulted in an amendment proposing her election as matron being carried by nine votes to three. In consequence of this decision Mr. Weston resigned the chairmanship of the Board. We rejoice that an attempt to enforce a religious test has been so successfully frustrated.

### VINDICATION OF A NURSE'S CHARACTER.

Last week an action brought by Margaret Ellen Ingle, a professional nurse, for libel against Dr. H. V. Bertram was tried in the King's Bench Division. The alleged libel consisted in a serious statement on the part of Dr. Bertram that Mrs. Ingle did not seem to be a fit person to have control of her patient, an old lady suffering from senile decay, and treated her brutally. This charge appeared from the evidence to have no further foundation than the fact that on a certain day when Dr. Bertram was present the patient became violently excited on hearing that some of her granddaughters had been called "forward little minxes," and the nurse, in order to soothe the old lady, put her arm round her and drew her away. It having been announced in court that Dr. Bertram withdrew the statements complained of in the action, and expressed regret for having made them, the plaintiff accepted the apology and a juror was withdrawn. Mrs. Ingle has thus obtained a complete public vindication of her character without pressing for damages.

### SHORT ITEMS.

A Presentation is to be made on Tuesday next to Miss Annie Hobbs, who for nearly three years held the post of Assistant to the Lady Superintendent of the Nurses' Co-operation. Tea will be served at the Howard de Walden Nurses' Home, from 4 to 6 for nurses on the staff who may be able to attend.—A much-needed home for the nurses attached to St. Olave's District Nursing Association was opened last week in Cherry Garden Street, Bermondsey.—The annual jumble sale in aid of Bridgend Cottage Hospital realised £98, and as the expenses only slightly exceeded £5 a substantial balance is at the disposal of the committee.

### Lectures to Nurses on Medical Nursing.

By Eveline A. Cargill, M.D.Brux., L.R.C.P.&S.Ed.

LECTURE X.—DISEASES OF THE KIDNEYS.—

*Continued from page* 54.

Diseases of the kidneys are accompanied by three distinctive groups of symptoms:—

1. Alteration in the quantity and composition of the urine. It is increased in chronic Bright's disease and diminished in the acute form. It always contains albumen, either much or little, and it sometimes contains blood.

2. Dropsy. This shows first in the eyelids, a puffiness under the eyes in the morning being often the first indication that anything is wrong with the kidneys; later there may be general dropsy. It is accompanied by a peculiar waxy whiteness of the skin, and in both these respects differs from the dropsy of heart disease, where the swelling begins in the feet and the skin has the blue tint due to venous congestion. In the dropsy of kidney disease the fluid which ought to be excreted by the kidneys accumulates under the skin. It is most marked early, when the skin is loose (eyelids), or in dependent parts, but soon becomes general in the abdomen and lungs and in the limbs, the arms being as much affected as the legs. When fluid accumulates under the skin it "pits on pressure"—the fluid is pressed out into the deeper parts and takes a little time to reappear; whereas healthy skin is elastic and resumes its rounded shape the instant the pressure is removed. The sign of "pitting on pressure" denotes the presence of fluid in the subcutaneous tissues, but not that the fluid is necessarily due to kidney disease.

3. Uræmia. By this we mean a group of nervous symptoms, produced by the absorption into the system of waste-products which ought to be excreted by the kidneys, but when absorbed they act as poison to the brain, and cause headache, twitching of muscles, breathlessness, vomiting, diarrhœa, etc., in chronic cases, or convulsions, coma or delirium in acute cases. Symptoms of uræmic poisoning always occur sooner or later in kidney disease, and, as a rule, the more violent the convulsions the better the chance of pulling the patient through; whereas in cases where uræmic symptoms come on very gradually and slowly the patient sinks into a comatose state from which he rarely recovers.

Various forms of eye disease are associated with affections of the kidneys. and the same rule applies to them; sudden blindness coming on after a convulsion passes away after a few days, but slow, gradual impairment of sight is permanent. Heart disease and kidney disease are closely associated. In advanced heart disease the kidneys are always affected, and in long-standing affection of the kidneys the heart becomes impaired, so that in seeing an advanced case for the first time it is often difficult to say if heart or kidneys are primarily at fault.

Acute nephritis comes on suddenly from a chill, say often after scarlet fever or diphtheria, and pregnant women are specially liable to it. It begins with a rigor, there is high fever, and the urine is scanty, high-coloured, and scalding. There is often pain in the loins of a dull-aching kind (pain is not a prominent symptom in kidney disease), and dropsy which quickly becomes general. The colour of the urine may be any variety of brown or red, smoky, porter-coloured or bright red. The redder it is (the more blood there is present) the more severe the inflammation. Albumen is always present, generally in large quantities, the urine on heating may become solid with albumen. Uræmic symptoms may soon develop with convulsions.

Chronic Bright's disease (so-called because Dr. Bright first connected the symptoms with disease of the kidney) is in a way a more serious disease than the acute form. It may follow an acute attack or come on gradually, but in either case the kidney substance becomes altered and destroyed, while acute nephritis is a pure inflammation, and is generally cured without any destruction of tissue. In the chronic disease as long as the urine continues abundant the poison is, to a large extent, washed out, but later, when the urine begins to fail, there is great danger. This form occurs in middle-aged people, chiefly men, and though incurable, much may be done to ameliorate symptoms and life may be prolonged for many years. Unfortunately the symptoms often come on so gradually that the disease may be far advanced before it is recognised.

In treating disease of the kidneys the aim is (1) to relieve the kidneys of work and (2) to get other parts of the body to take over some of the work. We cannot treat the kidneys directly, but we can diminish their work by cutting down nitrogenous food (as proteids are eliminated by the kidneys), therefore no meat is to be given or meat extracts, but a purely milk diet; and then we call in the assistance of the "complementary organs," *i.e.* skin and bowels, to remove water and some proteids by free purging (employing those drugs which produce watery evacuations), and encouraging the skin to act, by warmth, vapour baths, hot-air baths, and by drugs which produce perspiration. There is a peculiarly close relationship between the kidneys and the skin, the one is always ready to supplement or take on the work of the other. In cold weather, when the skin is dry, there is abundant urine; in warm weather abundant perspiration and scanty urine are associated. In uræmia when the kidneys secrete little or no urine the percentage of urea in perspiration is very much increased; that is, not only the quantity but the quality of the sweat varies with the condition of the kidneys, hence the immense value of skin action in kidney disease. The patient must be placed in a warm bed between blankets, wearing a well-aired flannel nightdress, and surrounded with hot-water bottles, or, better still, in a vapour bath. This may be improvised effectually by raising the bed-clothes over a cradle and inserting the spout of a bronchitis kettle into the space so formed, but the nurse must see that the steam does not play directly upon the patient's skin. The temperature of the room should be kept about 65°. The greatest care is required when attending to the patient to avoid any exposure of the surface, as the treatment renders it peculiarly sensitive, and a "chill" would probably be fatal. No utensil should be brought in contact with the patient without previously warming, and any change of dress or blanket must be carefully aired and put on warm.

When the most urgent symptoms have passed the same treatment must be persevered with, though somewhat modified. Warmth must be kept up but the patient should get out of bed as soon as possible to prevent œdema of the lungs. The bowels must be kept rather loose. The diet may be increased by fats and starchy foods, then fish or game, but no meat for a long time; indeed after acute inflammation of the kidneys meat is better withheld until the patient is practically well again. Alcohol should not be given unless by special orders.

In Dr. Cargill's Lecture on Diseases of the Kidneys which appeared on October 25, the sentence "young people are easily cured of diabetes" should have read "rarely cured."

### To Nurses.

We invite contributions from any of our readers, and shall be glad to pay for "Notes on News from the Nursing World," or for articles describing nursing experiences, or dealing with any nursing question from an original point of view. The minimum payment for contributions is 5s., but we welcome interesting contributions of a column, or a page, in length. It may be added that notices of appointments, entertainments, presentations, and deaths are not paid for, but that we are always glad to receive them. All rejected manuscripts are returned in due course, and all payments for manuscripts used are made as early as possible after the beginning of each quarter.

### Sketches of Our Hospital:—The Theatre.

BY E. MARGARET FOX.

It is operating afternoon to-day, and the theatre is ready for its work. It is a large, low, airy, many-windowed room, which at first sight suggests rather an artist's studio than an operating theatre. There are roof windows, screened just now by drawn white blinds, and side windows which, opening widely, give you a goodly view of a soft green lawn shimmering in the hot mid-day sunshine, with grand old trees sheltering little groups of convalescents, whose simple routine dinner is glorified by being eaten in the open, and a luxuriant kitchen garden and field beyond. Far away is a glint of a cool winding river, and belts of old forest trees. We are justly proud of the view from our theatre windows, and never fail to point it out to strangers who have climbed the many stone stairs leading to it, and are glad to pause awhile for breath before looking round. It is a quaint, unmodern room when you come to examine it. Curious attic beams cross it like a gymnasium; unexpected little doors swing open in the roof for ventilation, and when a gale is blowing, a very demon of wind whistles up through the lift shaft, and shakes the old-fashioned shutters that are like nothing so much as those you see outside a village shop in some forgotten hamlet.

A fearsome thing, known as a steam steriliser, stands in one corner, and breathes out threatenings and slaughter through many mouths ranged round its iron body at regular intervals, while it destroys the wily microbe lurking in the "dressings" and "sponges" cocking within its capacious bosom. A "pan, kettle, cauldron, or pot," but in plain English, a fish-kettle, bubbles away cheerily in another corner, suggesting approaching dinner to the uninitiated. Supported on thin iron legs, a high, narrow table occupies the post of honour in the best light, and wears a stern kind of uncompromising dignity as though it knows it is always the centre of attraction in that room of life and death. By some cunning contrivance, the table is constructed to hold hot water in its deep well to comfort with its genial warmth the poor martyrs stretched on it, and its india-rubber feet can be noiselessly and easily moved along, to facilitate its ready adjustment to the light required.

Running almost wholly down one side of the room is an important item in its furniture, and the most carefully tended and cared for. Those glittering steel things in that instrument case carry life and healing on their sharp and shining edges to many an unconscious victim as, wielded by the skilled hand of the surgeon, they wage war against disease, and wrest the germs of death from his unwilling grasp. In row after row they lie there in carefully-ordered groups, polished to dazzling brilliancy, clean and ready for instant use, on their shining shelves of glass. No court lady guards her diamonds with more thoughtful care than is accorded to these grim "jewels," for use though they are and not for ornament. For the rest, our theatre is bare and devoid of any unnecessary furniture. There is a curved glass-topped table, a few smaller tables, a cupboard or two, and some big jars and bottles. The floor is of dark polished wood, which needs restaining, and the paint of the walls and whitewash of the ceiling both want renewing. There is a lack of the latest up-to-date appliances and fittings that make the modern theatre such a curious and beautiful study in things scientific and convenient, and behind the lack of these things is an obvious lack of the "almighty dollar." Alas! that it should be so; that the hapless sufferers should be there in saddening numbers, the willing nurses, the able surgeons, yet often hampered and hindered in their earnest work by this same sordid need of money.

The warm sunshiny morning has merged into chilling rain and storm, and the drops chase one another mournfully across the many panes of glass. The theatre sister comes in to see if everything is at hand for the afternoon's work. She consults a thermometer, closes a window or two, tests the warmth of the hot-water table and of divers many-coloured lotions in bowls. Unlocking the instrument case she selects certain keen shining things from amongst them with great care, testing their edges and holding them up to the light. A tin case opens its mouth and yields up sundry bags, labelled "Sponges," but into which none may peep until the right moment comes for using them. There is a table full of significant articles covered by a white cloth, and there are white-lidded buckets, whose contents do not yet appear. The steam-steriliser has ceased to snort, but the "fish-kettle" is in particularly good form, ready to engulf those shining instruments and render them "aseptic" at a moment's notice.

A step on the stairs—quick, springy, purposeful, and one of the surgeons for the afternoon enters. He throws a comprehensive glance round the room, runs his eye over the instruments, suggests a few additions to them, puts a question or two to the theatre sister, and then divests himself of his coat. He is closely followed by others—anæsthetist, residents, on-lookers, until ten or twelve men begin to fill up the roomy floor space of the old theatre. A creaking and straining of a mighty rope, the obsolete shutters are unfastened, and the lift comes slowly up. There is space enough in it for a stretcher with its patient, the ward sister, and the lift attendant. The stretcher rolls noiselessly into the theatre, and practised hands quickly transfer its occupant to the operating table. She is a woman of about forty, her hands cold, and her face drawn with apprehension. She keeps her eyes tightly shut, as though to exclude the horrors she thinks must be around her, and she is trembling all over in an agony of nervous dread.

"Well, Mrs. Smith, how are you?" says the surgeon. "Come, open your eyes, there is nothing dreadful here."

The quiet reassuring tones of the surgeon give her confidence, and so does the warm human clasp of the theatre sister's hand upon her own poor trembling ones. She holds the sympathetic fingers tightly, and ventures to look about her, seeing for the most part only kind and familiar faces, and a complete absence of all knives or other horrors. She hears a voice at her head, saying: "Come, Mrs. Smith, don't be frightened; just smell this, and count after me one, two, and you will presently go to sleep and wake up in your own bed. Now count." She counts obediently one, two, three, four, and so up to twenty. The sister's clasp of her hands never loosens, and all is very quiet in the theatre. There is a strange dizzy feeling in her head, and once she feels as if she were being suffocated with that queer intoxicating smell from the innocent piece of flannel over her nose and mouth. She gasps for breath, and hears the sister saying in a far-away voice: "It's all right, Mrs. Smith. You are going on very nicely," and someone else says, quite in the distance: "Breathe away, go on counting, twenty-nine;" and she tries her best to obey, but somehow it is all confused, and she leaves off trying to do it, and forgets everything. Not till then does the sister loosen her hold of her hand, and remove sundry bandages, and "compresses" with the utmost celerity.

"She is ready now, sir," and presently the sister and nurse step back and give place to the surgeon and his assistant. It is all being done very quietly, very earnestly, and without an unnecessary word being spoken. The workers

### SKETCHES OF OUR HOSPITAL—*Continued.*

—doctors and nurses alike—are robed in long white overalls with bare arms and scrupulously clean hands. They look like priests and priestesses, performing high ritual at a sacrifice, so grave and reverent is their demeanour. The operation is a serious one, and grave risks attend it. Once or twice during its progress the anæsthetist looks anxious, and after feeling the patient's pulse, injects something into her arm from a syringe at his side. The operator pauses, and asks, "Is she all right?" and being reassured, works harder than ever. The "sponges"—which turn out to be little pads of wool wrapped in fine gauze—are given sparingly, and are constantly, unnecessarily counted, it would seem to a casual observer; but the initiated know full well what the result of an inaccurate count of those same innocent looking little sponges might mean to the helpless patient. In small packages of six they are doled out as they are wanted, and counted more than once during their use, the theatre sister, whose charge they are, wearing an anxious look until they are all returned to her safely. Willing fingers anticipate the surgeon's every want, and put into his hand each instrument as he requires it with as little delay as may be. The theatre clock ticks away the minutes loudly, and scarcely any other sound is heard except the heavy breathing of the unconscious patient. At last it is done. The surgeon stands back and mops his heated face with a sigh of relief. The patient's pulse is beating quietly, her breathing is good. He glances at the clock—time, thirty-five minutes—and goes to wash his hands again, elaborately.

"Next case, please," he says briefly.

The patient is returned to the stretcher, and the tension on everyone is removed. The surgeons change their overalls, and sit down at the open window, chatting over the details of the case. The theatre sister and nurse perform incredible feats of "cleaning up" in the short interval that elapses before the arrival of the next case. The rain has cleared off again, and brilliant sunshine is now on everything. Piercing screams from the lift, approaching rapidly nearer, and the next case, a small boy of eight, clinging with all his might to his ward sister, silences further attempts at conversation by his own terror-stricken cries. With difficulty is he got on to the table, and induced to lie down. The sight of those unfamiliar-looking white overalls fills him with dread, and his sharp eyes scent danger in the covered trays on the glass table.

"What's under them cloths?" he cries apprehensively, evidently suspecting treachery.

"See here, Tommy," says the anæsthetist, in his most seductive tones. "Like scent, do you? then just smell this nice stuff; now, there's a good boy!"

But Tommy is deaf to the voice of the charmer, charm he ever so wisely. He howls and kicks and screams until he finds that the wider he opens his mouth the more of that curious stuff goes down his throat, seemingly to choke him; so he sets his teeth, and fights for dear life as he thinks, till he gets so sleepy that he does not care to struggle any more; and then he is ready, being "under." Tommy's operation is not such a serious one as the last; but it takes longer, and when it is finished his head is swathed in many bandages, to which he will strongly object as soon as he comes "round." A baby of two years old is the next—a pretty, blue-eyed thing, who laughs up in the faces of those who bend over it, and who stuffs great handfuls of what it considers the edible portion of the anæsthetist's stethoscope into its mouth, and cries when it is removed. The wee arms and legs are warmly wrapped in cotton-wool, and as the little limbs rapidly become limp under the blessed influence of the chloroform, a hot-water bottle is put to the tiny feet, and warm towels are tucked round it. There are a few minutes of speechless anxiety during that operation, for once the feeble pulse nearly flickers itself out; the shallow breathing almost ceases, and the little face turns blue to the lips. But the danger passes, the baby shudders itself back to life again, and a loud cry is the sweetest music just then that the anxious onlookers care to hear. The last stitches are put in, and presently the ward sister gathers her precious baby into her arms and carries it back to the ward.

Briskly the clock ticks on, and the sun, from shining brightly in at one window, has crept on to another, and makes a perfect halo round the head of the surgeon. He does not appreciate halos, though, and asks curtly for the blind to be lowered. The temperature is rising and the theatre is very hot. There is rather a longer interval this time. The sister and nurse look flushed and tired, and the residents are heard to whisper something about "tea." Then, up comes the ponderous old lift again, and a powerful young navvy with his arm bandaged is brought in. Crushed by a terrible fall, the surgeons have been doing their best for weeks to try and save that arm but without success, and now amputation is the only hope left to him. He eyes his surroundings with no great misgivings as he sniffs the "inhaler," and at first is very quiet, responding most obediently to each direction, even grinning at the regulation question, "Any false teeth?" as he answers "No sir, not me!" But the theatre sister, who has met his kind before, mistrusts him, and quietly fastens a broad strap over his knees under the table, and arranges her instruments, watching him all the time. The strap is none too soon, for presently the young giant, under the influence of the intoxicating drug, rises in his strength, and requires four or five men to hold him down for the next few minutes. The native beauties of his everyday language—carefully controlled to his honour be it said in the ward—now appears in words unparliamentary in the extreme, terrifying the theatre nurse, who is new to her work, almost out of her wits. Every one is glad when he is finally overcome, and sinks into acquiescent stillness. Then, heat and fatigue being alike forgotten, the surgeons get to their work again with a will, and the poor, maimed right arm is lost to the young workman for ever. The sister's eyes are moist as she hands the bandages, and thinks of that fine frame, crippled for all time. But what will you? It was his last chance, and all that a man hath will he give for his life. There is added tenderness in the way the rug is tucked round him, when he is on the stretcher again, and the ward sister slips a pillow underneath to support the wounded shoulder on the way back to the ward.

It is nearly six o'clock now, and the operations for the day are all finished. Windows are thrown widely open, the lift shutters securely barred, reminding you more than ever of that little shop at closing time. The surgeons laugh and talk among themselves as they treat their hands to another detailed washing, and put on their coats once more. The residents whistle softly, and the theatre sister and nurse, who both look weary with the long afternoon, slip off their overalls, and begin to wash and clean instruments and bowls. For the work is not finished, when the operations are over and doctors are gone. Together, the sister and two nurses work on for more than an hour afterwards, mostly in silence, for they are too tired to talk. Every shining instrument must be duly washed, boiled, and replaced in immaculate order on its shelf. Every bowl, dish, and mackintosh must be left scrupulously clean. Lotion jars must be replenished, and everything arranged so that the theatre can be used again at a minute's notice, for no one can tell if some urgent case may not come in at dead of night, requiring instant operation. However, all is done at last, just as the sun is setting, and twilight coming on. The door is closed, and the soft evening air, blowing gently through the open window, gradually freshens the heated room. A typical operating day in our hospital is over!

### The Late Matron of the Royal Southern Hospital, Liverpool.

BY A CORRESPONDENT.

Miss Mary Gordon, whose retirement from the matronship of the Royal Southern Hospital, Liverpool, was announced last week, held that post most efficiently for nearly nineteen years. It is a very unusual, an almost unprecedented, event, to hear of three sisters retiring from such important posts at the same time—the matrons of St. Thomas's, Charing Cross, and the Royal Southern, Liverpool—or of so much good work having been done by one family of sisters for so many years, and all who know them will join in wishing that they may enjoy their well-earned rest.

To say of such a matron as Miss Mary Gordon that she gave great satisfaction to the committee is such an obvious truism that it seems superfluous to make the remark; the length of her services and the general efficiency of the hospital and staff speak for themselves, and those who worked with her longest know most how to appreciate her steady devotion to duty and to the best interests of those under her care. One special impression Miss Gordon seems to have left behind her as an example for others, namely, the wonderful power she possessed of entering into other people's feelings, the impartial justice with which she listened to each side of every dispute, and the unfailing interest with which she entered into the concerns of others, however full of business or worries she might be herself. During the many years Miss Mary Gordon worked in Liverpool her nurses and probationers have settled in every quarter of the civilised world, but wherever they may hear of her retirement they will think with respect and pleasure of the influence she had over them, and of the example she set them for their guidance in life. Miss Gordon's successor, Miss Sproule, who was trained at the Royal Southern, has been home sister and Miss Gordon's very efficient assistant for the last seven years.

### Everybody's Opinion.

**[Correspondence on all subjects is invited, but we cannot in any way be responsible for the opinions expressed by our correspondents. No communication can be entertained if the name and address of the correspondent are not given as a guarantee of good faith, but not necessarily for publication. All correspondents should write on one side of the paper only.]**

### QUEEN ALEXANDRA'S IMPERIAL MILITARY NURSING SERVICE.

"The Writer of Army Nursing, Past and Future," writes: May I be allowed to say in answer to "A Reserve Sister," whose excellent article appeared last week under the heading of "Everybody's Opinion," that in passing over the question of pay as needing "no comment" I did not intend to convey that it needed no reform, but that the alterations in the rate of pay did not seem to me of sufficient importance to need special mention in comparing the rules of the old and new services?

"Campaigner" writes: I entirely agree with all that "A Reserve Sister" says concerning the reorganisation of the Army Nursing Service, and think she shows herself possessed of more knowledge and common-sense than the civilians who are supposed to reorganise and brush away the "cobwebs" woven round the present system of Army nursing. On reading over the new "Rules and Regulations," it is my opinion that many of those who may be misguided enough to enter Q.A.I.M.N.S. as staff nurses will live to regret it, and may find that they could have done better in a civil appointment, where at least they are considered competent to measure poisons.

### VILLAGE NURSES IN CUMBERLAND.

"Miss L. Fletcher" writes: As a resident for many years in Cumberland, I have been interested in the correspondence which has lately appeared under the above heading in your valuable paper. My remarks are "without prejudice," as I have had no connection at any time with the Cumberland Nursing Association. I have watched its movements, however, with attention and interest; have read some of the Society's reports, and have made inquiries from friends who have had opportunities in their own neighbourhoods of watching the work done by the village nurses. I have no hesitation in stating my conclusion—viz., that the Cumberland Nursing Association is doing very valuable work among the rural and scattered districts of our (comparatively) poor and sparsely-populated country. Many of the criticisms made by your correspondents on the subject seem to me futile, for they were practically answered beforehand in Lady Lonsdale's excellent letter. Of course every lover of Cumberland would like to see a fully-trained nurse not only in some districts but in every parish. This ideal, alas! is impossible of attainment, as your correspondent "H. C. B." sensibly recognises. Lady Knightley writes of a successful nursing scheme near Daventry, where each district employs a fully-trained nurse at £80 per annum, the subscriptions being arranged so that "working people pay 4d. a month for each family, farmers and tradespeople 10s. a year, and others not less than £1." This suggestion again is entirely beside the mark as regards Cumbrian villages, where the chief subscribers classed as "the others" would be simply non-existent in many cases. As things are, poor folk have much reason in Cumberland to be thankful that the Nursing Association act on the common-sense principle that "half a loaf is better than no bread."

"R. D." writes: In last week's Hospital *Nursing Section* you state that I accuse the Cumberland Nursing Association of trying to drive me away from the district where I was working; that is not correct. I accused members of the local committee of trying their best to do so. Perhaps you are not aware that the local committee are formed amongst the parishioners of the districts, and are quite apart from the chief people of the Cumberland Nursing Association. Will you kindly make this right in your next issue?

### THE FUTURE OF THE ASYLUM NURSE.

"Dr. Urquhart" writes from James Murray's Royal Asylum, Perth: "An Asylum Worker" in her letter asks, what is to be the future of those of her fellow nurses who are trained and accredited as suitable and competent? That is a prominent question which should be considered by all who intend to make the nursing of the insane their profession. Not all can be matrons. Every assistant medical officer does not attain the rank of superintendent. But an efficient nurse has excellent chances of promotion. We need not consider the position of those who are placed in charge of wards, and decide to serve in that capacity until pensioned. The difficulty raised seems rather to be a certain preference shown for general hospital nurses in the selection of asylum matrons. As one of the first to indicate this preference, I may be permitted to make some reply, although it is now late in the day to argue the matter. The fact is, that asylums do not offer a wide enough field for the complete education of a matron, if it be held that a general practical knowledge of bodily diseases and accidents is essential to the position. Would it be desirable, even if possible, that any asylum committee should appoint a medical superintendent, whose knowledge of his profession had been solely gained in a special hospital for the insane? Nurses who desire to become matrons must qualify for these posts, not only in the care of all classes and conditions of the insane as taught in modern institutions, but also in the wards of general hospitals and obstetric clinics. Do asylum physicians ask too much in this respect? I think not. In the first place, every matron ought to have proved her capabilities as a nurse, and in the second place she ought to be qualified to undertake the duties of her position in the care of all kinds of patients, and in the instruction of the nurses in every detail. One hears of asylums where it is claimed that their nurses are thoroughly trained and fully qualified after a course of three years' instruction. I utterly deny that any asylum is competent to turn out nurses with the highest professional claims to appointments as matrons. Indeed it was urged by those who carried their point in limiting the course of asylum instruction for the M.P.A. certificate to two years, that a longer term would be a hardship to those who desired to proceed to general nursing work. The result is a course of five years or thereby—the same length of time as is required for the medical profession, but under very different conditions as to payments. No doubt the organisation of the nursing profession will be accomplished before long, but it seems strange that the general hospitals do not follow the example of the asylums and combine to regulate the training of nurses, and secure to those who are properly qualified the position to which they are entitled. My experience, generally, has been that it is better for a nurse to begin in an asylum and thence to pass to a general hospital, where her position as a person of skill and suitability is duly recognised and where promotion for her is speedy. Now that nursing is less of a fashionable foible and more of a real profession, hospital matrons are glad to secure the services of asylum nurses and not likely to keep them long at unprofitable probationer work. I only regret that male attendants are not similarly received for training in general hospitals so that they might similarly advance in their profession. The "higher berths" in asylums have been given to general hospital nurses because, as a rule, they have lately shown higher qualifications for the duties; but, so far as I can discern the signs of the times, that side door to promotion will soon be closed. It would be absurd to suppose that the partial education of a general hospital nurse would be accepted as equivalent to the full training of an asylum nurse who has completed her apprenticeship in a general hospital. The claims of the asylum nurse thus fully qualified must be paramount. Means are provided therefore for enabling the asylum nurse to become fully proficient, and the woman who will adopt these means is on the high road to promotion. I have no reason to complain of the "material" of the nursing staff, but one may regret that the average duration of service is shortened by these new ideals. The best nurses go on to complete their education, and thereafter find plenty of scope for their energies in life. Looking over a recent list I find that nine nurses have left us to enter general hospitals, and four have gone to nursing institutions. When the time comes for these nurses to apply for matronships in asylums I have little doubt of their success in commanding favourable consideration, because they have been proved competent not only in a specialised branch of nursing, but also in the general work of their profession.

---

"Charles H. W. Bridgeland" writes from Hants County Asylum, Knowle, near Fareham: I was greatly pleased at reading "Asylum Worker's" interesting letter in The Hospital to discover that there are still some among us who feel only too keenly that the nursing of the insane does not receive its due amount of recognition when compared with the other branch of the profession. I am sure it cannot be ascribed to want of ability, because, speaking from my own experience, I consider the training received in the infirmary ward of an ordinary public asylum is quite equal, if not superior, to that received in many of our large general hospitals and infirmaries. In my opinion the remedy to this evil lies to a great extent with the authorities, and I am afraid that little can be done without their co-operation. In the first instance, narrow the age limits for both male and female attendants, and appoint them on the condition of their signing a contract to serve for a stated period as probationers. I am confident that this would go a long way towards eliminating those "undesirables" who take a position as asylum nurse or attendant as a stopgap till some more congenial employment turns up. Again, make it compulsory for them to enter for the examination held under the auspices of the Medico-Psychological Society, and I may state here that it would be desirable to make the examinations more severe and to enter more into the general details of the other branches of the profession. If these few suggestions were ever given a trial, I am confident that none would entertain the idea of starting life as an asylum attendant without a real liking for the work, and that an asylum-trained male nurse would be on a far superior footing to what he is at present, and in process of time would be looked upon as an essential part of the staff in all our large general hospitals. I differ with "Asylum Worker" as to the question of the charge of the infirmary ward in an asylum. She evidently thinks it of common occurrence for the authorities to go outside of the asylum when a "charge" is wanted to look after the sick under their care. It may be done in a few isolated cases, but as a rule, he or she, is selected from their own staff, or from that of another asylum. To those who have studied the nursing of the insane, it is quite evident that a sick nurse, more often than not, would be practically useless. We all know that an infinite amount of tact is sometimes needed in dealing with a sane person when stricken down with illness, who is very peevish and irritable. How much more then is needed in nursing the insane, many of them homicidal and suicidal cases and needing constant observation? I am afraid that the average hospital nurse would be in very sore straits if put in charge of the infirmary ward of any of our asylums and told that she would be held responsible for anything that happened while the ward was under her care. In conclusion I may state that those who have charge of the insane are as a rule well versed in all branches of nursing, and, if not, it is entirely their own fault, because every facility is offered one to obtain a thorough insight into the work. As a general rule, too, the medical officers are always most thoughtful in rendering every assistance to nurses or attendants whom they see are anxious to make their mark in the walk of life they have chosen.

---

### Appointments.

[No charge is made for announcements under this head, and we are always glad to receive, and publish, appointments. But it is essential that in all cases the school of training should be given.]

Bromley Cottage Hospital.—Miss Florence A. Harris has been appointed senior staff nurse. She was trained at Newport and Monmouth Hospital, where she was afterwards staff nurse and theatre sister.

Canterbury Hospital.—Miss Minnie Dix has been appointed night sister. She was trained at Edinburgh Royal Infirmary, and has since been ward sister at Hull Royal Infirmary, and ward sister at St. Mary's (Islington) Infirmary, Highgate Hill, N.

Homœopathic Hospital, Birmingham. — Miss Louisa Mold has been appointed sister of the children's wards and theatre. She was trained at the Royal Infirmary, Edinburgh, and has since done private nursing for two and a half years for the Private Nursing Institute, Burton-on-Trent, and for the last two years has held the position of theatre sister at the Stanley Hospital, Liverpool.

Hospital for Diseases of the Throat, Golden Square, W.—Miss K. E. Whitehead has been appointed matron. She was trained at Charing Cross Hospital. She afterwards became sister of the children's medical and surgical wards, and subsequently sister of the men's medical ward. For nearly two years she has held the post of night superintendent in the same hospital.

Manchester Children's Hospital, Pendlebury.—Miss Sarah Louise Loughton has been appointed lady superintendent. She was trained at St. Helen's Hospital and St.

Thomas's Hospital, London, where she was afterwards sister and night superintendent. She was also sister (*pro tem.*) at the Nightingale Home.

Portsmouth Workhouse Infirmary. — Miss Edith D'Arcy has been appointed charge nurse. She was trained at Hackney Workhouse Infirmary.

Queen Victoria Royal Infirmary, Preston.—Miss Kathleen Bowyer has been appointed sister. She was trained at Manchester Royal Infirmary, and has since been theatre sister in the same institution, sister in the children's ward and night sister at Warrington Infirmary, and deputy matron at the City Hospital North, Liverpool.

### Presentations.

Charing Cross Hospital —Miss K. E. Whitehead, who is leaving Charing Cross Hospital to take up the duties of matron at the Throat Hospital, Golden Square, W., has been presented by the staff of sisters and nurses with silver-mounted hair and hat brushes, silver-mounted tortoise-shell comb, trinket tray, etc.

### Novelties for Nurses.

By Our Shopping Correspondent.

### FIRE-PROOF GARMENTS.

All nurses, and district nurses more particularly, are sensible to the danger to helpless patients arising from fire. This danger is especially emphasised in the case of old and bed-ridden patients who are left much to themselves, and whose imperfect sight the close proximity of a light render necessary. In such cases a material called Non-flam. Safety Flannelette will prove of much service, and should be universally recommended. I would suggest that the entire bed and personal clothing of bed-ridden patients might most advantageously consist in winter of this flannelette. There are varieties of colour and thickness which render it adaptable to all requirements. Flannelette sheets are not irritating, as woollen ones might prove, and they are quite inexpensive. Another direction in which nurses might with advantage introduce the flannelette to notice is amongst the children of the poorer classes, whose predilection for playing with fire is notorious, and who run continual risks from accidents when not under a controlling observation. The flannelette is manufactured by Messrs. Whipp, of Aytoun Street, Manchester, and it can be ordered through any linendraper. In London it is obtainable at Messrs. Whiteley, and Swan and Edgar. Later Messrs. Whipp will apply their process to all kinds of cotton materials, and then nurses will have an opportunity of securing greater safety by adopting a "safety" uniform.

### "PIRLE" FINISHED MATERIALS.

Nurses purchasing Pirle finished materials are asked to look for the word "Pirle" which is woven into the selvedge of all genuine Pirle finished materials. Worthless imitations have been introduced of the specialities finished by Messrs. Ripley and Son, of Bradford, and purchasers are warned to examine materials for the trade mark.

### Where to Go.

Dowdeswell Galleries, 160 Bond Street.—Mr. Oliver Hall is showing at the Dowdeswell Galleries 40 cabinet pictures in oil for a season commencing on November 5th.

### For Reading to the Sick.

### HE KNOWETH BEST.

Among so many, can He care?
Can special love be everywhere?
A myriad homes,—a myriad ways,—
And God's eye over every place?

I asked: my soul bethought of this;—
In just that very place of His
Where He hath put and keepeth you,
God hath no other thing to do!

*A. D. T. Whitney.*

Gratitude is the loving recollection of those who, in some sense, "first loved us."

And has not God a first call on this? Count up your blessings; perhaps they are so familiar to you, so strongly secured to your possession by what seem, from habit, indissoluble bonds, that you have forgotten that they are blessings. Better at once awake from that dream. "All flesh is grass, and all the glory of man as the flower of the grass. The grass withereth, the flower fadeth." Perhaps you have nourished the habit of looking at the dark side till it has the power, the tenacity, of second nature. Look at the bright side now.

Think of the blessings you have in common with all. The sun that shines "upon the evil and the good"; the rain that "falls upon the just and the unjust"; "creation, preservation, and all the blessings of this life." Are these too "common," too part and parcel, as you deem it, of the indefeasible rights of humanity? Well, think of particular mercies—your home, your friends, the dear souls whose presence is your sunniest day, whose absence is your darkest cloud. Think of yourself, your health, your strength; it may be your recovery from serious sickness. Think of the many pleasant days that have been; such an evening in the thirsty, glowing summer; such a morning in the early spring; such a pleasant country walk; such a still night on the moonlit sea; the vast world of beauty and glory which broke upon your spirit from the memories and the thoughts of the mighty dead, from the magnificence of nature, above all, from the warm, pure, tender, unfailing affection of the human heart.

Our happiness is a very secret thing, but a very real. A heart may agonise, while yet, with calm countenance, it bravely faces life. A heart, too, amidst the commonest toils, the most distasteful duties, may have (which may not, if it will?)—may have, from the inner and outer treasures that it holds, an unfailing spring of pure happiness. Who gave it? Recognise the Giver.—*Knox Little.*

What channel needs our faith, except the eyes?
God leaves no spot of earth unglorified;
Profuse and wasteful, lovelinesses rise;
New beauties dawn before the old have died.

Trust thou thy joys in keeping of the Power
Who holds these changing shadows in His hand;
Believe and live, and know that hour by hour
Will ripple newer beauty to thy strand.

*T. W. Higginson.*

### Echoes from the Outside World.

### The King in London.

The King upon his return to London on Monday visited Wyndham's Theatre, and on Tuesday inspected the battalion of the Brigade of Guards which arrived in town last week too late to join the review. His Majesty wore the uniform of Colonel of Scots Guards, and was accompanied by Lord Roberts who was attired as Colonel of the Irish Guards. The ceremony took place in front of Buckingham Palace, and was witnessed by a large number of persons. The King having expressed the great satisfaction he felt at seeing the battalion after their long spell of active service in South Africa, congratulated them upon their return and upon the high state of their efficiency. At the conclusion of his speech three cheers were enthusiastically given, at the instance of the Colonel of the battalion for his Majesty. As the representatives of the press could not, from the terrace of the Palace, hear the King's speech, a staff sergeant of the Scots Guards took a short-hand note which he afterwards read aloud for their edification.

### War Honours.

A long list of war honours was published on Saturday, the most interesting, perhaps, being the bestowal of a G.C.B. on Lord Methuen, who the previous day had a great public reception at Devizes. Lord Lansdowne in proposing the health of the guest at the luncheon, said that "there was no officer in the Army who bore from the beginning to the end a heavier share of the stress and burden of the war, nor had any officer borne that burden more steadfastly and more cheerfully than Lord Methuen, whether fortune was kind or unkind to him." Major-General M. W. Willson has been made a K.C.B., Sir John French and Sir Ian Hamilton become Lieutenant-Generals, and Colonels Kekewich and Plumer Major-Generals. The dignity of Knight-bachelor has been conferred upon Colonel James Gildea of the Soldiers and Sailors Families' Association; Mr. Frederick Harrison, General Manager of the London and North-Western Railway, and Mr. Charles Owens, General Manager of the South-Western Railway, for their services at home in connection with the war. Another new Knight is Mr. George Farrar, who was condemned to death after the Jameson raid. When the war broke out he put himself at the disposal of the military authorities, and did good service.

### Mr. Chamberlain's Visit to South Africa.

The course of the armoured cruiser *Good Hope*, in which Mr. Chamberlain is to start on the 24th or 25th to South Africa, is to be by the East Coast route *viâ* the Suez Canal, and Durban will probably be the port of landing. The Colonial Secretary has, with great regret, decided that the time at his disposal will not be sufficiently long to render it practicable for him to include Rhodesia in his tour. There is some difficulty in arranging the details of the banquet to be given on the eve of Mr. Chamberlain's departure, because the number of applications for tickets is so great that it is difficult to secure a hall with sufficient accommodation. Anyhow ladies will not be permitted to be dinner guests. Mr. Chamberlain's own constituents intend to have a gigantic demonstration after the banquet with a torchlight procession, and probably 5,000 torch-bearers will line the route.

### Conference of Women Workers.

The National Union of Women Workers had a successful meeting in Edinburgh last week. Lady Battersea presided, and amongst the papers read was one by Miss Frances Melville, Warden of University Hall, St. Andrews, and another by Miss A. M. Sellar, late vice-principal of Lady Margaret Hall, Oxford. Miss Melville, speaking for Scotland on University Education, said that the matriculation of women students had increased from 260 in 1892 to 947 in 1901. Miss Sellar, for England, stated that the happiness of women, especially of single women, had been greatly increased by higher education. There were many more possibilities for them, and there was greater freedom from meaningless, conventional restrictions. In that change she recognised two main factors—university education and the bicycle. The question was often asked whether the higher education had diminished the number of marriages. She admitted that the number of university women who married was not great. She firmly believed, however, that the right sort of marriage was not prevented by anything so irrelevant as education, but rather that true education fitted a woman for married life. Lady Frederick Cavendish and Lady Frances Balfour discussed women's suffrage. Lady Frederick said she favoured women serving on local boards, but Imperial politics she should leave alone. Lady Frances, on the other hand, affirmed that she did not believe the sky would fall if women sat in Parliament. Wage-earning of children was also a subject of discussion. Some members were in favour of totally prohibiting casual employment outside school hours; others wished for wise regulation rather than prohibition; and one lady stated that the home life was being stamped out by the School Board giving home lessons. Another interesting point which was ventilated was the case of the feeble-minded. Miss Dendy, of Manchester, maintained that it was probable that two-thirds of the crime of the country might be prevented by a scientific method of dealing with the feeble-minded. Hooligans, criminals, paupers, and drunkards were frequently what they were because their minds were feeble. A scheme of industrial colonies should meet the need. Discussions upon the Education Bill and upon the advisability of making publicans discontinue the employment of women in bars concluded the meeting.

### Mrs. Humphry Ward's Play.

Considerable interest attached to Mrs. Humphry Ward's first appearance as a dramatist, and "Eleanor," which is being performed at the Court Theatre matinées—where it is played every afternoon but Wednesday—is much discussed. The drama is adapted from the novel by the authoress herself, and it suffers, as do most plays of this kind, from the fact that it is difficult for the playwright to forget that she is also a novelist. Here, however, blame ends. Some of the scenes are both forcible and beautiful. That in the second act, when the mad woman, Alice Manisty, attempts to take the life of the young American girl, Lucy Foster, is vivid and effective; and that in the third act, when the two women who love the same man, confide in each other, is a most clever psychological study. The part of Eleanor appears as if it had been created entirely for Miss Marion Terry, so finely does she embody it. Her acting at first is so restrained and quietly dignified that it renders the scenes when she "lets herself go" the more striking. This clever actress has never done anything better. Much praise, too, must be accorded to the Alice Manisty of Miss Elizabeth Robins, and the innocent tender grace of Miss Lilian Braithwaite's Lucy Foster. A vivacious French lady, Madame Variani, is played as crisply as might be expected by Miss Rosina Filippi. The men are rather heavily handicapped by Mrs. Ward. The hero, Edward Manisty, is such an unattractive person that it is impossible for Mr. Charles Quartermaine to make one believe he could have been loved by two women. Mr. Bernard Fox as Father Benecke is a disappointment, through no fault of his own, but Mr. Leslie Faber, as Reggie Brooklyn, is able to do something to brighten the scenes where he appears. The dresses of the actresses, especially Miss Terry's, are both lovely and in excellent taste.

### Notes and Queries.

**The Editor is always willing to answer in this column, without any fee, all reasonable questions, as soon as possible.**

**But the following rules must be carefully observed:—**

**1. Every communication must be accompanied by the name and address of the writer.**

**2. The question must always bear upon nursing, directly or indirectly.**

**If an answer is required by letter a fee of half-a-crown must be enclosed with the note containing the inquiry, and we cannot undertake to forward letters addressed to correspondents making inquiries. It is therefore requested that our readers will not enclose either a stamp or a stamped envelope.**

*A Midwife's Duties.*

(34) Can adrenalin be given in douches for p. p. hæmorrhage, and would a midwife be allowed to use it in this way?—*Queen's Nurse.*

What a midwife will be allowed to do will depend upon the decision which the Midwives' Board arrive at on this subject, and no doubt when this Board is constituted the limitations to be imposed upon a midwife's duties will receive careful attention. It would seem *primâ facie* that the duty of the midwife should be to attend to healthy women in normal confinements, and that wherever abnormality occurs—or, in other words, whenever it becomes a question of treating illness—a doctor should be sent for. In attending confinements, however, a midwife must be prepared to deal efficiently with accidents, and of all accidents post-partum hæmorrhage is the most urgent, and the one demanding the most immediate attention. But we think that where a midwife finds herself called upon to deal with an emergency like this, she ought not to experiment, but should follow strictly, and with the greatest care, the exact methods which she has been taught. Now the use of adrenalin may be a very good thing; it is undoubtedly a very effective mode of stopping certain forms of bleeding; but the teaching of the schools and of all experience is that the only satisfactory mode of controlling post-partum hæmorrhage is to induce contraction of the uterine muscle, or pending the occurrence of such contraction to actually grip the uterus between the two hands. If the experience of our great maternity charities should in the future show the use of adrenalin to be a better plan, then, of course, in an emergency midwives will have to adopt it—indeed it will then, no doubt, be taught to them in their training schools; but till then they will certainly be going out of their way, will be exceeding their duty in one direction, and sadly neglecting it in another, if they omit the use of methods they have been taught for the sake of employing speculative drug treatment.

*Reading Union.*

(35) I have been told that an American Reading Union exists which recommends various medical books and sets them papers on nursing subjects. Are there any such correspondence classes in England, and if not, can you give me the address of the secretary of the American Reading Union?—*Queen's Nurse.*

We do not know the address of the secretary of the American Reading Union, but the editor of the *Trained Nurse*, Metropolitan Building, New York, might be able to give it you. A reading union has been tried here, but, as Miss Moberly, the originator, announced a few weeks ago, it did not obtain sufficient support.

*Enteric Fever.*

(36) In classifying, for purposes of reference, the cases of illness treated in a nursing home, would it be usual or in accordance with the best up-to-date opinions to rank enteric fever under the heading "infectious diseases"?—*Non-professional.*

Certainly. Enteric fever is one of the diseases included in the list of "infectious diseases" given in the Infectious Diseases (Notification) Act, 1889.

*France.*

*Army Nurse.*

(38) 1. I should be very glad if you would tell me how to become an Army nurse. 2. Is there any other military hospital besides the one at Netley?—*Gwladys.*

1. Send for a form of application to the Matron-in-Chief, Queen Alexandra's Imperial Military Nursing Service, Horse Guards, S.W. 2. There are several military hospitals. We published in our issue of October 18th a full copy of the regulations of Queen Alexandra's Imperial Military Nursing Service, in which the Army Nursing Service will be merged.

*America.*

(39) Can you give me any information with regard to private nursing in America? Will you also give me the name of any institution or hospital?—*H. P.*

Articles on private nursing in America frequently appear in our columns, and a list of American hospitals is published in "Burdett's Hospitals and Charities."

*Home.*

(40) Can you kindly tell me of one or two good nursing homes in the north of London where I could receive comfort and good nursing during my confinement which takes place at the end of November?—*G. F.*

A short advertisement would probably bring you many replies from suitable homes if none of those advertising in our columns fulfil your requirements. Why not consult your medical man?

*Diabetes.*

(41) Can you tell me where to get a list of foods suitable for a patient with diabetes? and also where I can get a work on the subject?—*B. H.*

The Manager of the Scientific Press would on application send you a list of the latest books on the subject. One or two cookery books have given special attention to the preparation of food for this complaint. That by Miss Maude Earle is excellent.

*Lunacy Laws.*

(42) I have a mental patient living with me under a receiving order from the Lunacy Commissioners. Will you kindly tell me if I could receive another uncertificated case without asking their permission? Their visitor has always been very good to me, and I should not like to do anything illegal.—*A. A.*

It is permissible, but it may save misunderstanding if you were to write to the Visitor of the Lunacy Commission and explain.

*Scarlet Fever.*

(43) Will you kindly tell me the most infectious stage of scarlet fever. I have always been taught that it is during the peeling stage, but a doctor who is also M.O.H. for the country district where he lived, emphatically denied that there was any infection, excepting during the rash and throat period?—*E. W.*

Some medical men hold opinions at variance with those generally accepted. Scarlet-fever patients require isolation for two months, or longer, if desquamation continues.

*Translations.*

(44) I should be obliged if you could tell me where I could get German medical works to translate. I am a maternity nurse, and understanding German I wish to turn to account my spare time.—*M. A. J.*

Translations are very difficult to get, and badly paid when obtained. You might possibly get some work from a medical man privately by advertising.

*Queen's Nurse.*

(45) Will you kindly let me know how to become a Queen's nurse. Am trained, and have been a nurse 12 years? Do Queen's nurses only do district work?—*G. A. H.* and *Nurse L.*

Yes; Queen's nurses look after the sick poor only in their own homes. Write to the General Superintendent, Queen Victoria's Jubilee Institute for Nurses, St. Katherine's Precincts, Regent's Park, N.W., for full particulars.

*Afloat.*

(46) I am a nurse with three-years' certificate, and have as well fever and maternity training. I wish to obtain the position of nurse to first-class passengers on board one of the big liners. Will you kindly tell me to whom I should apply in order to get such an appointment?—*K. C.*

You can only apply to the secretaries of such companies as provide nurses on their vessels, and they are very few and far between. You might also advertise in the shipping journals.

**Standard Books of Reference.**

**"The Nursing Profession: How and Where to Train." 2s. net; post free 2s. 4d.**

**"Burdett's Official Nursing Directory." 3s. net; post free, 3s. 4d.**

**"Burdett's Hospitals and Charities." 5s.**

**"Hospital Expenditure: The Commissariat." 2s. 6d.**

**"The Nurses' Dictionary of Medical Terms." Cloth, 2s.; leather, 2s. 6d. net.; post free, 2s. 8d.**

**"Burdett's Series of Nursing Text-Books." 1s. each.**

**"A Handbook for Nurses." (Illustrated). 5s.**

**"The Physiological Feeding of Infants." 1s.**

**"The Physiological Nursery Chart." 1s.; post free, 1s. 3d.**

**All these are published by the Scientific Press, Ltd., and may be obtained through any bookseller or direct from the publishers, 28 and 29 Southampton Street, London, W.C.**

### Travel Notes.

**By Our Travelling Correspondent.**

### CXIII.—THE PEARL OF BRIESGAU.

It is getting late in the season for short holidays, but there are those who, having been compelled to give precedence to their elders and betters, are obliged to take their well-earned rest late in October and even in November; I should like to draw the attention of such belated holiday-makers to Freiburg in the Black Forest, affectionately called by its inhabitants "the Pearl of Briesgau."

### Cost of the Journey and Living.

The cheapest way of all is by Harwich, Antwerp, and Strassburg, and costs, second-class return, £4 13s. 9d. The other way, by the Hook of Holland, takes you through Heidelberg and costs 4s. more. There are several good, comfortable hotels where you may live for 5s. per day, and probably less by arrangement. It is not a smart town in any way, and mercifully it is still fairly free from tourists, so that prices are not exorbitant and one sees a German town in its natural state and not devoured with a desire to cater for hordes of casual travellers.

### Its Suitability for a Late Holiday.

I have been there several times, but my last visit was in a sunny cold October, and it struck me then as being a nice little place for those whose duties compelled a late flitting. There is but little to occupy the evenings naturally, and that is always a little difficult to find unless you visit some gay city such as Paris or Brussels, or go much further afield.

There is, however, a good theatre open from September to May and an opera too, opening at the same time but closing at Easter. Then there are frequent concerts and a fine military band, which plays at various places. Your landlord will give you full particulars. It is a great military centre, which is ever a joy to me; that element always adds gaiety, life, and picturesqueness to any town, and the German soldier is a beautiful person to whatever branch of the service he belongs.

One reason why Freiburg is so pleasant in the late autumn is because there are many easy excursions to be made around, notably to the Hollenthal, and being a richly-wooded country the tints are positively superb.

I drove to Freiburg from Titisee straight through the Hollenthal, or Valley of Hell; but why this exquisite gorge should have so gruesome a name I know not, except that in places the opening is very narrow and consequently it is dark. You can take the rail all through this beautiful part, and you see it quite as well as by driving, the two tracks running parallel, but the nicest way is to take train to one of the little stations early in the day (provided with some luncheon), wander about exploring the country and returning when daylight wanes. By doing this to different stations on various days, we thoroughly traversed that most beautiful locality.

### The Munster.

It stands in a most picturesque square, which on market day presents a delightful vista to the artist or photographer. The wealth of colour massed together in the fruit and vegetable stalls is rivalled by the stands of cheap but serviceable drapery, the whole presided over by comely women of all ages, mostly with handkerchiefs of various tints on their heads and round their ample shoulders.

I like much to come upon the cathedral from the Kaiser Strasse just opposite an ancient and most beautiful fountain. In front of the west door are three columns surmounted by statues of the Blessed Virgin, St. Lambert, and St. Alexander, and high above tapers the openwork spire, which somewhat resembles that of Strassburg. The effect of the evening sun seen through this from the public gardens is very striking.

Our hotel was close to the munster. We chose it so as to be able to run in and out when we liked, and to sketch in the market-place when the spirit moved us. The pulpit alone is worth taking a long journey to see; it is an exquisite gem of the early sixteenth century. I spent an entire afternoon endeavouring to get a satisfactory remembrance of it. It curls lovingly round one of the mighty pillars, and the lines are charmingly graceful. There are some fine stained windows of the fifteenth century, chiefly in the south aisle. The rose window is also old, but the others in the north aisle, etc., are modern.

Opposite the south portal stands the Kaufhaus, a fifteenth century building, very picturesquely placed; inside here is a fine staircase, not generally shown, but I caught a glimpse of it through an open door.

Freiburg is really a delightful city; I do not know what inspires the feeling, but it seems a friendly place, it is not too large and above all it is not infested by trippers. I loved to wander up and down the main street called the Kaiser-Strasse, look at the shops and study the pedestrians; it is a very pretty street, though mostly modern, except for the grand old Martinsthor, a massive gateway with a delightfully realistic fresco of St. Martin sharing his cloak with the beggar.

There is another gateway of the same character called the Schwabenthor, it stands in the Salz-strasse, and has a fresco of a Suabian peasant with a wine waggon. The Rathhaus and the old University are both interesting; there is a curious courtyard and staircase at the back of the former. I had no time for making a sketch, but succeeded, by squeezing myself flat in a neighbouring doorway and standing on a pail, in securing a photograph.

Such articles as offered for sale in the town are of the poorest description. One of the distinctive features of the place is the number of fountains, ancient and modern, but all picturesque; then, too, water perpetually flows clear and sparkling down conduits through all the principal streets; these conduits are crossed in front of each house door by a little stone bridge. It gives a very bright, clean effect to the town.

### Excursions to be Made.

Besides the Hollenthal there are numerous delightful walks or cycle trips to be made. There is that to Ludwigshöhe, reached by passing through the Suabian gate and mounting the Schlossberg.

Then the Jägerhäusle, or huntsman's hut, half an hour's walk from the suburb of Herdern. There is a good refreshment place, and it is pleasant to wander out there for afternoon coffee.

St. Ottilien, a walk of an hour and a quarter.

Further off, and requiring some five or six hours, lie the Kybfelsen, the Kukuksbad, and the Rosskopf, and still further afield, needing the devotion of an entire day, lie Schaninsland, the Feldberg, and many other spots.

### TRAVEL NOTES AND QUERIES.

Aix-les-Bains (E. L. T.).—I wish my kind correspondents would use a pseudonym. I hope, however, that this will catch your eye. The addresses and information you give are really useful. The terms for good accommodation at Aix are very reasonable, and I much value the testimony of personal experience. You are right, both places are Villes de Luxe.

